# Elucidation of an Allosteric Mode of Action for a Thienopyrazole RORγt Inverse Agonist

**DOI:** 10.1002/cmdc.202000044

**Published:** 2020-03-12

**Authors:** Rens M. J. M. de Vries, Richard G. Doveston, Femke A. Meijer, Luc Brunsveld

**Affiliations:** ^1^ Department of Biomedical Engineering and Institute for Complex Molecular Systems Eindhoven University of Technology Den Dolech 2 5612 AZ Eindhoven The Netherlands; ^2^ Leicester Institute of Structural and Chemical Biology and Department of Chemistry University of Leicester University Road Leicester LE1 7RH UK

**Keywords:** Nuclear Receptors, RORγt, Allosteric Modulators, Structure Elucidation, Drug Discovery

## Abstract

The demand for allosteric targeting of nuclear receptors is high, but examples are limited, and structural information is scarce. The retinoic acid‐related orphan receptor gamma t (RORγt) is an important transcriptional regulator for the differentiation of T helper 17 cells for which the first, and some of the most promising, examples of allosteric nuclear receptor modulation have been reported and structurally proven. In a 2015 patent, filed by the pharmaceutical company Glenmark, a new class of small molecules was reported that act as potent inverse agonists for RORγt. A compound library around the central thienopyrazole scaffold captured a clear structure‐activity relationship, but the binding mechanism of this new class of RORγt modulators has not been elucidated. Using a combination of biochemical and X‐ray crystallography studies, here the allosteric mechanism for the inverse agonism for the most potent compound, classified in the patent as “example 13”, is reported, providing a strongly desired additional example of allosteric nuclear receptor targeting.

## Graphical abstract

**Example 13**: Inhibition of the RAR‐related orphan receptor gamma t (RORγt) nuclear receptor has proven to be a novel approach for targeting autoimmune diseases. Recently, RORγt was shown to feature an allosteric pocket. Using X‐ray crystallography, we now show that compound **13**, a promising thienopyrazole analogue of previously reported RORγt modulators, exerts its inverse agonism by binding to this allosteric pocket.

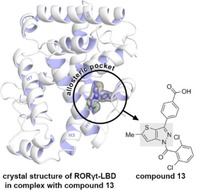

## Introduction

Nuclear receptors (NRs) are a family of ligand‐dependent transcription factors that constitute an important class of drug target.^1^, ^2^ Within this family, the retinoic acid‐related orphan receptor gamma t (RORγt) has garnered much attention as an intervention point to treat autoimmune diseases such as multiple sclerosis, psoriasis and inflammatory bowel disease. A significant characteristic of these diseases is the excessive production of the pro‐inflammatory cytokine interleukin 17 (IL‐17) by Th17 cells. RORγt plays a key role in Th17 cell differentiation. Disruption of the IL‐17 signaling pathway using recently FDA‐approved monoclonal antibodies already demonstrated to be an effective strategy for the treatment of psoriasis.^3^, ^4^, ^5^ Therefore, inhibition of RORγt would be a highly promising alternative therapeutic strategy.^6^, ^7^, ^8^ Numerous small molecules have been reported that effectively inhibit RORγt, which is transcriptionally active even in the absence of any endogenous agonist.^9^, ^10^, ^11^ Typically, such inverse agonists bind to the orthosteric NR binding pocket; a pocket conserved across many NRs, but with concomitant challenges in achieving NR subtype selectivity and competition with endogenous ligands. Allosteric modulation of NR activity offers a promising novel concept for addressing such challenges and for novel modes of NR drug action in general.^12^, ^13^, ^14^ However, examples of structurally characterized and sufficiently potent NR allosteric modulators are scarce. The discovery and development of such compounds is therefore urgently needed in order to steer their molecular design process and further our conceptual understanding of allosteric NR modulation.^13^

**Figure 1:**
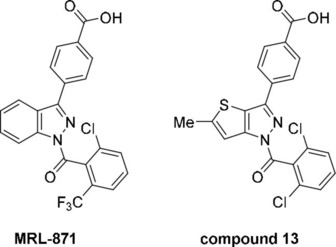
The chemical structures of allosteric RORγt inverse agonists **MRL‐871** (MSD) and compound **13** (Glenmark).

Recently, allosteric modulation of RORγt was shown to be a promising approach for drug development,^15^, ^16^, ^17^ featuring examples of allosteric ligands with high NR subtype selectivity and absence of competition with the endogenous ligands.^18^ The first example to emerge was the indazole **MRL‐871** (Figure 1), a potent inverse agonist. Originally disclosed by Merck Sharp and Dohme in 2012,^19^ its allosteric mode of action was characterized three years later.^15^ Glenmark Pharmaceuticals used the **MRL‐871** core as the basis for an *in silico* scaffold hopping screen in search of a novel but similarly potent compound class.^20^ This led to the development of a family of thienopyrazole inverse agonists with nanomolar activity which were disclosed in a 2015 patent, where ‘compound **13’** was the most potent example (Figure 1).^21^ Although a comprehensive structure–activity relationship (SAR) study was conducted around the thienopyrazole scaffold, the binding mode to RORγt was not reported.

**MRL‐871** and **13** only differ with any significance in their central heteroaromatic core (Figure 1). Due to the similarity between the two compounds, we postulated that the thienopyrazole series shared the same allosteric binding mode as indazole **MRL‐871**. Here, we report X‐ray crystallography and match this with biochemical binding data, confirming an allosteric mode of action for the exemplar thienopyrazole **13**. The structural characterization of new classes of allosteric RORγt inverse agonist is of great value in terms of better understanding SAR with respect to this allosteric pocket. Furthermore, it contributes to our growing conceptual understanding of NR allosteric modulation in general.

## Results and Discussion

**Figure 2:**
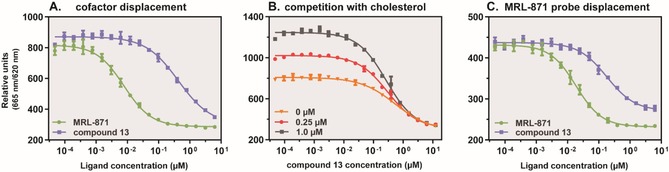
Bio‐chemical assay data for **MRL‐871** and compound **13**. (A) Dose‐response curves from the TR‐FRET coactivator recruitment assay for **MRL‐871** and compound **13**; (B) Dose‐response curves from the competitive TR‐FRET coactivator recruitment assay for compound **13**, with fixed concentrations of cholesterol (0 μM, 0.25 μM and 1.0 μM); (C) Dose‐response curves from the ligand displacement TR‐FRET assay for **MRL‐871** and compound **13**, using a fluorescently labelled MRL probe. Data adapted from Meijer *et al*. 2020.^16^

In recent work to develop novel allosteric RORγt inverse agonists we reported the synthesis and inverse agonistic behavior of compound **13**, which was used as a reference molecule.^16^ In a time‐resolved FRET (TR‐FRET) coactivator recruitment assay we reported that compound **13** effectively inhibited coactivator peptide binding, and thus the background constitutive activity of RORγt, in a dose‐dependent manner with an IC_50_ value of 425±61 nM (Figure 2A).^16^ This was less potent than indicated in the original patent disclosure where an IC_50_ value of <50 nM was reported using a similar assay format.^21^ In agreement with previously reported values, **MRL‐871** was found to be ∼50 times more potent (7.8±0.5 nM). To determine if compound **13** has an allosteric mode of action analogous to **MRL‐871**, we performed a competition TR‐FRET assay. Compound **13** titrations were performed in the presence of increasing concentrations of the well‐characterized orthosteric agonist cholesterol (Figure 2B). Consequently, the addition of cholesterol stabilizes the active conformation, thereby increasing the FRET signal in the absence of compound **13**. The binding curves revealed that the presence of the agonist does not lower the inhibitory potency of **13**, but instead even slightly enhances it (IC_50_=269±19 nM in the presence of 1.0 μM cholesterol).^16^ The absence of competition between cholesterol and **13** suggests that the binding mode of **13** is independent to that of cholesterol and therefore that **13** likely binds to an allosteric pocket on RORγt. Next, we sought evidence to prove that compound **13** was indeed binding to the same allosteric site as **MRL‐871**. For this, we performed a ligand displacement assay using a previously reported **MRL‐871**‐derived fluorescent probe that is known to bind the previously published allosteric site. Compound **13** effectively displaced the probe in a dose‐dependent manner (Figure 2C).^16^ These results are thus a strong indication that compound **13** binds to this specific allosteric pocket. Nevertheless, probe displacement could also be caused by changes in protein conformation induced by binding of compound **13** binding to another site on RORγt.

**Figure 3:**
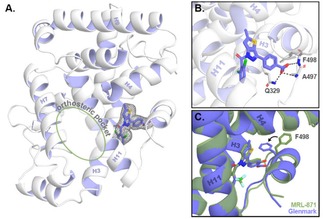
Co‐crystal structure of RORγt with compound **13** (PDB code: 6TLM). (A) The tertiary structure. The final 2Fo−Fc electron density map around compound **13** is shown as an isomesh contoured at 1σ; (B) Close‐up of the allosteric pocket. The polar interactions between RORγt and compound **13** are shown as a grey dotted line. (C) Overlay of the crystal structure of RORγt bound to compound **13** (blue) and RORγt bound to **MRL‐871** (green; PDB: 5C4O).

**Figure 4:**
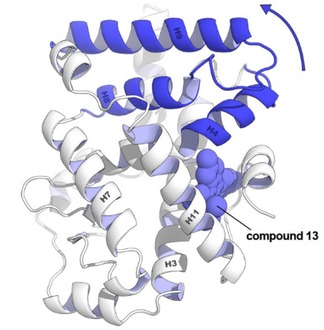
Conformational change in the tertiary structure of RORγt comparing the complex with compound **13** and **MRL‐871**. The methyl substituent on the thienopyrazole increases the bulk toward helix 4. The helices that change position compared to the **MRL‐871** structure are coloured dark blue and are moving in the arrow's direction.

To now unambiguously confirm the binding mode of compound **13**, we determined the structure of the **13**‐RORγt binary complex using protein X‐ray crystallography. A C455H mutant of the ligand‐binding domain (LBD) of human RORγt was used for this study; the region surrounding this native cysteine is important for crystal packing of RORγt in complex with allosteric inverse agonists.^15^, ^16^ RORγt and compound **13** were co‐crystallized and crystals grew as hexagonal bipyramids overnight using sitting‐drop vapor diffusion. The data collection and refinement statistics of the crystal are provided in Table S1. The crystal structure (PDB: 6TLM) shows the complete LBD of RORγt and reveals that compound **13** indeed binds in the anticipated allosteric pocket and that the orthosteric pocket is devoid of ligand (Figure 3). Of specific and notable interest is the folding of helix 12, in a conformation precluding coactivator binding. The carboxylic acid of **13** interacts with Q329 and two backbone amides of A497 and F498 located in the loop between helix 11 and 12 of RORγt. The binding mode of **13** to RORγt is comparable to that of **MRL‐871**. A first notable difference in the binding to RORγt of both ligands lies in the increased bulk of compound **13** toward helix 4 of RORγt, resulting from the methyl substituent on the thienopyrazole core. This induces a shift of helix 4, correlated with a displacement of helix 9 (Figure 3C and Figure 4). A second, and most pronounced, difference can be observed in the loop between helix 11 and 12. Compared to **MRL‐871**, the benzoic acid moiety of compound **13** is orientated differently in the pocket, which is connected to a change in the overall fold of this loop. In particular, F498 changes conformation and thereby flips away from helix 4. A comparable behavior is observed for the recently documented RORγt allosteric inverse agonist **FM26**, where a pyrrole introduces bulk toward helix 4 (Figure S1).^16^ Both **13** and **FM26** induce a fold of the loop between helix 11 and 12 that has F498 in the “flipped‐out” conformation. In contrast to compound **13**, the pyrrole of **FM26** makes an additional interaction with the backbone carbonyl groups of L353 and K354, which can explain the higher affinity of **FM26** for RORγt (IC_50_=264±23 nM).^16^

Comparison of the IC_50_ values of compound **13**, **FM26** and **MRL‐871** would suggest that steric bulk toward helix 4 does not favor the affinity of the allosteric modulator for RORγt (Figure S1). MSD's patent application showed that bulky substitutions on the 4 and 5 position of the indazole moiety also resulted in a reduced affinity for the receptor.^19^ Glenmark did disclose the less bulky hydrogen‐substituted thienopyrazole, but the biochemical activity of this analog was not evaluated. However, various compounds were evaluated incorporating bulkier amide substituents replacing the methyl group.^21^ Such compounds generally showed a lower or no binding affinity for RORγt.

## Conclusions

In summary, using X‐ray crystallography and supported by biochemical studies, Glenmark's compound **13** was shown to bind to an allosteric pocket of RORγt. The binding mode of compound **13** is similar to **MRL‐871** and the recently reported **FM26**, with small but notable differences. The structural data imply that the lower affinity of **13** for RORγt relates to additional bulk on the thienopyrazole core pointing to RORγt helix 4, which leads to changes in the overall protein fold. Such changes are likely to affect the dynamics of the protein and stability of the specific fold. The new structural data expand the collection of crystallized ligands binding to allosteric binding pockets on NRs, and specifically on RORγt. The resulting new insights will aid in the understanding of reported compounds classes, potentially also addressing the same allosteric RORγt pocket and in the development of new compound classes with more diverse chemotypes or optimized pharmacodynamics profile.

## Experimental Section

### General

The synthesis of compound **13** and the biochemical assays have been described earlier.^16^

### RORγtC455H‐LBD expression and purification for crystallography

The expression and purification of RORγtC455H‐LBD were performed as described earlier.^16^ In short, a pET15b vector encoding for RORγt LBD (AA 265–507) incorporating a C455H mutation was transformed into E. Coli BL21 (DE3) cells. These cells were cultured in 2x YT medium supplied with 0.05 % antifoam SE‐15 (Sigma Aldrich) with 100 μg/mL ampicillin. After an OD600 of 0.6 is reached, protein expression was induced by adding 0.25 mM IPTG. The protein expression continued overnight at 15 °C. Centrifugation was used to collect the cells which were lysed using an Emulsiflex‐C3 homogenizer (Avestin). The resulting solution was purified using Ni‐NTA affinity chromatography. The elution fraction was dialyzed overnight in buffer A without imidazole and thrombin was added to remove the purification tag. The purified sample was subsequently purified using gel filtration. Fractions containing RORγtC455H were collected and concentrated before being flash‐cooled and stored at −80 °C.

### X‐ray crystallography

Compound **13** was dissolved in 50 % DMSO and 50 % EtOH to a final concentration of 20 mM. One equivalent of compound **13** was added to the RORγt protein solution and the mixture was placed on ice. After 1 hour incubation, centrifugation at 20.000 RCF for 20 minutes at 4 °C was used to remove protein and ligand precipitate. Sitting‐drop vapor diffusion was used to generate crystals using 800 nL of the protein‐ligand solution and 400 nL crystallization solution (1.6 M (NH_4_)_2_SO_4_ and 0.1 M Tris (at pH 8.5)). Crystals grew as hexagonal bipyramids to their final size overnight. Because the crystallization solution was not cryogenic, the crystal was briefly transferred to a cryo‐solution (1.6 M (NH_4_)_2_SO_4_, 0.1 M Tris, 25 % glycerol and 200 μM compound **13** (at pH 8.5)) before being flash‐cooled. The crystal was measured at the i24 microfocus beamline of the Diamond Light Source (Oxford, United Kingdom). Initial data processing was performed using the CCP4i2 suite (version 7.0.078).^22^ DIALS (2.0.2) was used to integrate the data.^23^ Because the diffraction was anisotropic, STARANISO was used to correct the data.^24^ Aimless was used to scale the corrected data.^25^ Using the RORγt crystal structure in complex with allosteric ligand **FM26** (PDB: 6SAL) as a search model for molecular replacement, PHASER was used to phase the data and ligand restraints were generated using AceDRG.^16^, ^26^, ^27^ REFMAC and COOT were used for sequential refinement and model building.^28^, ^29^ Final refinement was performed using phenix.refine from the Phenix software suite (version 1.16_3459).^30^, ^31^ Figures were made with PyMOL (version 2.2.3, Schrödinger).^32^

## Conflict of interest

The authors declare no conflict of interest.

## Supporting information

As a service to our authors and readers, this journal provides supporting information supplied by the authors. Such materials are peer reviewed and may be re‐organized for online delivery, but are not copy‐edited or typeset. Technical support issues arising from supporting information (other than missing files) should be addressed to the authors.

Supplementary
