# Supplementary material for: Elucidation of an Allosteric Mode of Action for a Thienopyrazole RORγt Inverse Agonist
Source: ChemMedChem. 2020 Mar 12;15(7):561–5. doi: 10.1002/cmdc.202000044 (PMC7187189; doi:10.1002/cmdc.202000044)
Supplement: Supplementary file 1 — Supplementary [file CMDC-15-561-s001.pdf]

CHEMMEDCHEM

Supporting Information

Elucidation of an Allosteric Mode of Action for a Thienopyrazole RORγt Inverse Agonist

Rens M. J. M. de Vries, Richard G. Doveston, Femke A. Meijer, and Luc Brunsveld*© 2020 The Authors. Published by Wiley-VCH Verlag GmbH & Co. KGaA. This is an open access article under the terms of the Creative Commons Attribution License, which permits use, distribution and reproduction in any medium, provided the original work is properly cited.

Table S1. Data collection and refinement statistics for the crystal structure of RORγt in complex with Glenmark's compound **13**.

RORγt in complex with compound 13	
Data collection	
Space group	P 6_1 2 2
Cell dimensions	
a, b, c (Å)	107.528 107.528 98.737
α, β, γ (°)	90, 90, 120
Resolution (Å)	93.12-2.32 (2.40-2.32)
I / σ(I)	6.9 (0.8)
Completeness (%)	100.00 (100.00)
Redundancy	35.9 (36.5)
$CC_{1/2}$	0.992 (0.422)
Refinement	
No. unique reflections	15190 (1448)
R_{work}/R_{free}	0.2054/0.2520
No. atoms (non-H)	
Protein	2020
Ligand	28
Water	23
Average B-factors	
Protein	58.86
Ligand	52.19
Water	57.94
R.m.s. deviations	
Bond lengths (Å)	0.016
Bond angles (°)	2.024
PDB ID	6TLM

WILEY-VCH

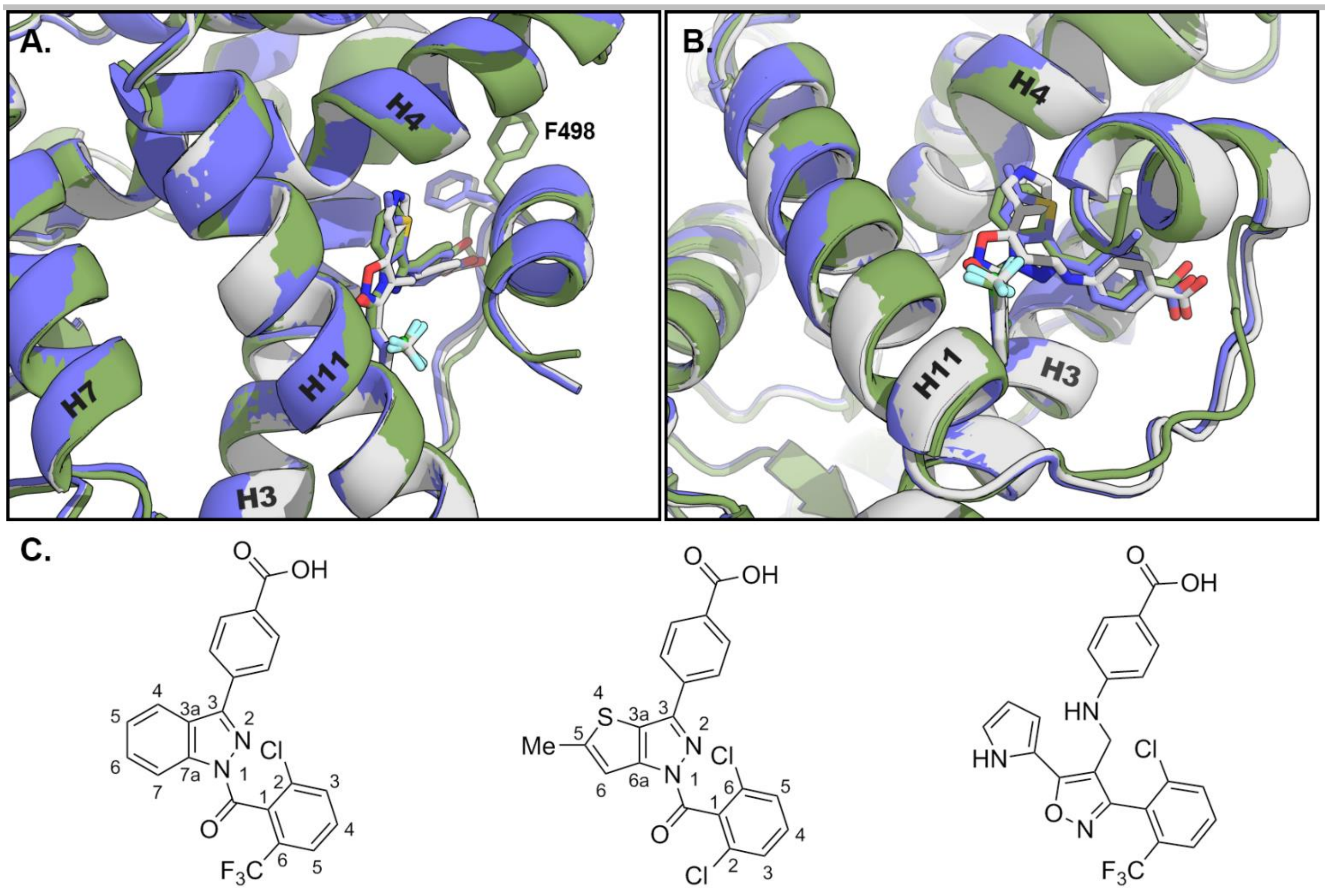

MRL-871
IC_{50} = 7.8 ± 0.5 nM

compound 13
IC_{50} = 425 ± 61 nM

FM26
IC_{50} = 264 ± 23 nM

Figure S1. (A-B) Crystal structure comparison of RORγt in complex with **MRL-871** (green, PDB: 5C4O), **FM26** (white, PDB: 6SAL) and compound **13** (blue, PDB: 6TLM). Compound **13** and **FM26** induce the same fold of the loop between helix 11 and 12, by the interaction between the benzoic acid on the ligand and F498 of RORγt. (C) Chemical structures of **MRL-871**, compound **13** and **FM26** showing the IUPAC atom numbering. The IC_{50}-values of the ligand-RORγt complexes were measured previously using a TR-FRET coactivator recruitment assay.[16]

WILEY
